# Correlates of Psychological Well-being Among Korean Vietnam War Veterans

**DOI:** 10.1093/geroni/igab046.3273

**Published:** 2021-12-17

**Authors:** Hyunyup Lee, Sungrok Kang, Soyoung Choun, Carolyn Aldwin

**Affiliations:** 1 Korea Military Academy, Seoul, Seoul-t'ukpyolsi, Republic of Korea; 2 Oregon State University, Corvallis, Oregon, United States

## Abstract

Prior research on Veterans’ mental health has largely focused on identifying risk and protective factors for negative psychological symptoms such as PTSD. However, mental health indicates not merely absence of psychopathology, but also the existence of positive psychological well-being (Keyes, 2005). Thus, the current study aimed to examine the correlates of psychological well-being, which is less studied, in an Asian sample, Korean veterans. Data for this 2017 study were from Korean Vietnam War Veterans Study. Participants were 348 male veterans, and their mean age was about 72 years old (SD = 2.7, range = 65-84). Using Keyes’ (2002) classification criteria, psychological well-being was divided into three types: flourishing (9.5%), moderately health (59.95%), and languishing (25.3%). Own-way analyses of variance showed that the groups did not differ in demographic variables (age, marital status, education, and income). Further, there were no differences in combat exposure, negative appraisals of military service, smoking, and alcohol consumption. However, significant group differences were found for resources; Scheffé's post-hoc analyses indicated that optimism, positive appraisals of military service, four types of social support (family, significant others, friend, and military peer), and self-rated health were significantly different among the groups, and highest in the flourishing group. The moderately health group showed higher levels of positive appraisals of military service and four types of social support than the languishing group. Thus, the majority (about 60%) of Korean Vietnam veterans were moderately psychologically healthy in this sample, but those with positive psychosocial resources were more likely to be healthiest.

